# The effects of acute renal denervation on kidney perfusion and metabolism in experimental septic shock

**DOI:** 10.1186/s12882-017-0586-6

**Published:** 2017-05-31

**Authors:** Emiel Hendrik Post, Fuhong Su, Koji Hosokawa, Fabio Silvio Taccone, Antoine Herpain, Jacques Creteur, Daniel De Backer, Jean-Louis Vincent

**Affiliations:** Department of Intensive Care, Erasme Hospital, Université Libre de Bruxelles, Route de Lennik 808, 1070 Brussels, Belgium

**Keywords:** Acute kidney injury, Sepsis, Septic shock, Renal blood flow, Renal function

## Abstract

**Background:**

Perfusion deficits likely play an important role in the development of renal dysfunction in sepsis. Renal denervation may improve kidney perfusion and metabolism.

**Methods:**

We randomized 14 female sheep to undergo bilateral surgical renal denervation (*n* = 7) or sham procedure (*n* = 7) prior to induction of sepsis. Renal blood flow (RBF) was measured with a pre-calibrated flowprobe. Laser Doppler probes were implanted to measure cortical and medullary perfusion. Cortical glucose, lactate and pyruvate levels were measured using the microdialysis technique. Creatinine clearance was determined. Sepsis was induced by peritonitis and fluid resuscitation was provided to avoid hypovolemia.

**Results:**

RBF and cortical perfusion were higher in the denervated group during the first 6 h after induction of sepsis (*P* < 0.001 and *P* < 0.05, respectively), while medullary perfusion decreased similarly in both groups. After hypotension developed, RBF decreased to similar levels in both groups. Cortical pyruvate and lactate levels were lower in the denervated animals (*P* < 0.001 and *P* < 0.001, respectively). There were no differences between groups in creatinine clearance, urine output or time to oliguria.

**Conclusion:**

Denervation thus caused an early increase in RBF that was distributed towards the kidney cortex. Although associated with an attenuation of early cortical metabolic alterations, denervation failed to prevent the deterioration in renal function.

## Background

Sepsis and septic shock are frequently complicated by acute kidney injury (AKI) [1–3] but its underlying pathogenesis remains poorly understood and therapeutic options are limited [4]. Several experimental studies have indicated a potential association between perfusion deficits and renal dysfunction in endotoxemia and sepsis [5–8], but others have demonstrated increased renal blood flow (RBF) in septic AKI, suggesting a limited role for hypoperfusion and tissue hypoxia in the development of sepsis-associated renal dysfunction [9]. Most human studies have shown a reduction in relative RBF, or fraction of cardiac output directed to the kidneys, indicating that perfusion-metabolism mismatch may occur [10–12]. Attempts to manipulate RBF in sepsis have, however, been largely ineffective [13, 14].

While the importance of increased renal sympathetic outflow is well-established in the context of chronic arterial hypertension [15, 16], it is less clear whether elevated renal sympathetic nerve activity (RSNA) is equally relevant in acute disease [17]. In a rat model of endotoxemia, Wang et al. found that chronic renal denervation protected kidney function, an effect that was likely mediated by the preservation of RBF [18]. Conversely, in an ovine model of hyperdynamic sepsis caused by infusion of live bacteria, chronic denervation had no effect on RBF or kidney function [19]. However, acute, as opposed to chronic, renal denervation results in an immediate increase in RBF that may be sustained for several weeks or more [20, 21]. This immediate, but potentially transient, effect of renal denervation may be of particular value in early sepsis, as many of the factors contributing to the need for renal denervation will disappear once sepsis resolves.

Given the controversial role of RBF in sepsis-associated AKI and the acute effects of renal denervation on renal perfusion, we designed a study to evaluate the effects of acute renal denervation in a large animal model of septic shock. We hypothesized that acute renal denervation would augment renal perfusion in sepsis and attenuate the occurrence of renal dysfunction.

## Methods

We studied 14 adult female sheep (laboratory-owned domestic animals, aged 8–10 months, weight 28 [24–33] kg) that were fasted for 18 h prior to the start of the experiment with free access to water. The animals were deeply sedated with intravenous ketamine, midazolam and morphine throughout the experiment and were observed until spontaneous death. The ethical committee of the Free University of Brussels approved the study, and handling of the animals was in accordance with the ARRIVE guidelines [22].

### Anesthesia and ventilation

The animals were premedicated with an intramuscular injection of 0.5 mg/kg midazolam (Dormicum; Roche SA, Anderlecht, Belgium) and 40 mg/kg ketamine (Imalgine; Merial, Lyon, France). A 14G peripheral cannula was introduced into the cephalic vein to provide vascular access. The animals underwent endotracheal intubation following induction of anesthesia with 30 μg/kg fentanyl citrate (Janssen Pharmaceutica, Beerse, Belgium) and 0.5 mg/kg of rocuronium bromide (Esmeron, Organon, Oss, The Netherlands). Sedation and analgesia during the surgical procedure were achieved using midazolam at a rate of 1.5 mg/kg/h, ketamine hydrochloride at 10 mg/kg/h, and morphine at 1.0 mg/kg/h. Mechanical ventilation was performed in volume-controlled mode with tidal volumes of 10 mL/kg and PEEP set at 5 cmH_2_O. The respiratory rate was adjusted to maintain PaCO_2_ values between 35 and 45 mmHg. Fraction of inspired oxygen (FiO_2_) was set at a value of 30% and adjusted to keep PaO_2_ > 80 mmHg. A 60-cm tube was inserted into the stomach to drain its content and left in situ to prevent rumen distension. A 14F Foley catheter (Beiersdorf AG; Hamburg, Germany) was introduced into the bladder to monitor urine output (UO).

### Surgical preparation

The left carotid artery was surgically exposed and a 4.5 F arterial catheter (Vygon; Cirencester, England) was introduced to enable blood sampling and monitoring of the arterial pressure. A 7F introducer was inserted into the right jugular vein and a pulmonary artery catheter (CCO; Edwards LifeSciences Corp., Irvine, California) advanced under monitoring of waveforms. A midline laparotomy was then performed. The cecum was punctured and feces were extracted and stored at room temperature. The cecum was closed and returned to the abdominal cavity. The abdomen was closed in two layers and a tube was inserted through the abdominal wall for later injection of feces. The animals were then turned to the prone position. A bilateral flank incision was performed and the renal artery was identified on both sides. A flow probe (PS series 6 mm; Transonic, Ithaca, NY) was carefully positioned around the left renal artery.

### Experimental protocol

**Fig. 1:**
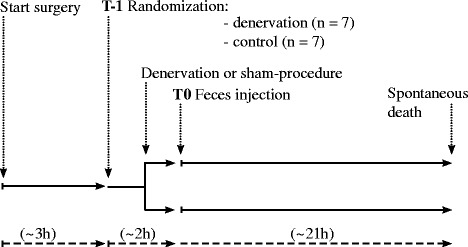
Flowchart of the experimental protocol

After placement of the flow-probe, the animals were allowed to rest for approximately 1 hour after which the first baseline measurements were taken (T-1, Fig. 1). The animals were then randomized to either bilateral denervation (denervation, *n* = 7) or sham procedure (control, *n* = 7) using the envelope method. Renal denervation was performed by surgically stripping the renal artery from its adventitia and applying 20% phenol in 95% alcohol solution for 15 min while carefully avoiding damage to the surrounding tissues [19, 23]. An immediate increase in RBF was considered evidence of successful denervation [18, 19]. During the sham procedure, the adventitia was left untouched and the artery was moistened with a 0.9% NaCl-solution.

A 3F catheter was then introduced into the renal vein using the Seldinger technique. The kidney fascia was carefully punctured and a microdialysis catheter (membrane length 4 mm, cut-off 20 kDa; CMA 20; Microdialysis, Solna, Sweden) was introduced into the renal cortex. A second puncture was made to insert a laser Doppler probe (OxyFlo; Optronix, Oxford, United Kingdom) to enable cortical perfusion measurements. A final puncture provided access for the insertion of a laser Doppler probe into the renal medulla. To avoid traumatic injury by dislocation, the probes were inserted after surgical denervation and, therefore, no Doppler measurements were taken at T-1.

All variables were then measured (T0) before injection of 1.5 g/kg of autologous feces into the abdominal cavity to induce peritonitis [24, 25]. Fluid resuscitation consisted of 6% hydroxyethyl starch (HES) (Voluven; Fresenius Kabi, Schelle, Belgium) and Ringer’s lactate (Baxter SA, Lessines, Belgium) given in a 1:1 ratio and titrated to PAOP. The maintenance rate was set at 4 mL/kg/h and fluid challenges of 100 mL Ringer’s lactate solution and 100 mL HES 6% were given if PAOP fell below its baseline value. When SV increased more than 10%, the fluid infusion rate was increased by 2 mL/kg/h [24, 25]. If MAP decreased to <65 mmHg in the absence of fluid-responsiveness, shock was considered refractory [26] and fluid infusion was set at a constant rate of 10 mL/kg/h. Since hypoglycemia commonly occurs in this sheep model, we monitored blood glucose concentrations by arterial blood gas analysis and attempted to keep these at baseline levels via the administration of boluses of 50% glucose.

### Hemodynamics and general monitoring

Cardiac output and heart rate (HR) were monitored continuously. Mean arterial pressure (MAP) and pulmonary artery balloon-occluded pressure (PAOP) were obtained at end-expiration. Hourly arterial and mixed venous blood gas samples were taken to measure pH, hemoglobin (Hb, g/dL), monitor plasma glucose (mg/dL) and mixed venous oxygen saturation (SvO_2_; %), enabling the calculation of the systemic oxygen delivery index (DO_2_I; mL/min/m^2^), oxygen consumption index (VO_2_I; mL/min/m_2_), oxygen extraction ratio (O_2_ER; %) and the veno-arterial PCO_2_ difference (P_v-a_CO_2_; mmHg). P/F ratio was defined as the ratio of partial pressure of arterial oxygen to FiO_2._ Cardiac index (CI; L/min/m^2^) and systemic vascular resistance index (SVRI; dynes·s/cm^5^/m^2^) were calculated according to standard formulas. Body surface area (BSA) was calculated for each animal according to a previously published formula [27].

### Renal hemodynamics and kidney function

Left RBF (mL/min) was recorded hourly and the ratio of RBF to cardiac output, or relative RBF, was calculated (RBF/cardiac output; %). Renal DO_2_I, renal VO_2_I and renal O_2_ER were also calculated according to standard formulas. Renal vein blood was aspirated carefully to avoid contamination with inferior vena cava blood. Laser Doppler values were measured continuously in arbitrary blood perfusion units (BPU). UO was assessed hourly. Every 3 hours, a one-hour urine collection was obtained for measurement of urine creatinine and sodium content. In addition, a blood sample was taken for determination of plasma creatinine (mg/dL) and plasma sodium concentrations (mmol/L). Creatinine clearance (mL/min), filtration fraction (FF, %), sodium reabsorption (TNa^+^, mmol/min) and fractional excretion of sodium (FENa, %) were calculated according to standard formulas.

### Assessment of renal cortex metabolism

The microdialysis catheter was perfused with a dedicated perfusion fluid (CMA T1; CMA Microdialysis AB; Na^+^ 147 mmol/L, K^+^ 4 mmol/L, Ca^2+^ 2.3 mmol/L, Cl^−^ 156 mmol/L, osmolality 290 mOsm/kg). The perfusion-rate was set at 0.3 μL/min (miniaturized infusion pump CMA107; CMA Microdialysis AB). The time needed for adequate equilibration depends on the metabolites of interest and is thought to be between 60 and 120 min [28]. We chose to collect the perfusate every 60 min in microvials, after a one-hour stabilization and washout period. Samples were analyzed for lactate (lactate_cortex_, mmol/L), pyruvate (pyruvate_cortex_, μmol/L) and glucose (glucose_cortex_, mg/dL) by a bedside analyzer (CMA600 Microdialysis Analyzer; CMA Microdialysis AB). The lactate/pyruvate ratio (cortical L/P ratio) was calculated as lactate_cort_/pyruvate_cort_ × 1000. The mean cortical L/P ratio for all animals in which it has been measured in our lab is 18.9 ± 6.9 and we therefore considered values of 32.7 or more (mean + 2 × SD) as abnormal. The gradients between plasma and interstitial lactate (ΔLactate_cortex_) and pyruvate (ΔPyruvate_cortex_) were calculated by subtracting plasma from interstitial levels for lactate and pyruvate, respectively.

### Measurement of plasma epinephrine

Blood samples were collected every 6 h, placed on ice and centrifuged at 5000 rpm for 10 min. The supernatant was immediately collected and stored at −80 °C. Plasma epinephrine concentrations (μg/L) were determined using a high-performance liquid chromatography electrochemical detection technique [29]. This technique has a limit of detection of 0.06 μg/L and a limit of quantitation of 0.19 μg/L.

### Measurement of plasma pyruvate

Plasma pyruvate (μmol/L) was determined every 6 hours. Blood samples were immediately deproteinized by the addition of 8% perchloric acid (Sigma Aldrich, Machelen, Belgium) and centrifuged at 8000 rpm. The supernatant was then stored at −20 °C. Plasma pyruvate levels were measured using the enzymatic-UV method with linearity between 30 and 450 μmol/L and a measurement error of 3.7% [30].

### Statistical analysis

Data were truncated at 18 h and are presented as mean ± standard deviation. Linear mixed effects models were used to test for the main effects of group and time and their interaction. When a significant effect of time was observed, a Student’s paired t-test was carried out to compare variables at each time-point with their respective baseline values. In case of significant interaction between group and time, Student’s t-test was performed to compare the groups at each time-point. Time to event analyses were carried out using a Log-Rank test and are presented as median and [range]. Two-sided tests were used and a *P*-value of less than 0.05 was considered statistically significant. Analyses were performed using the R statistical data environment (R, version 3.1.1; R Coreteam, Vienna, Austria).

## Results

### General findings and systemic hemodynamics

The two groups had similar baseline variables before randomization (T-1, Table 1). At T0, CI had increased transiently and SVRI decreased in the denervated animals compared to their values at T-1. MAP decreased similarly in both groups throughout the experiment and time to hypotension, defined as MAP <65 mmHg, was also similar (Table 2). PAOP was adequately maintained in all animals throughout the experiment and, after induction of sepsis, CI remained at baseline levels in both the denervation and control groups. The amount of fluid administered was similar in the two groups. The P/F ratio decreased similarly in both groups. Plasma epinephrine increased similarly in both groups (Table 3). Systemic lactate and pyruvate concentrations were not significantly different in the two groups at any time point. Survival times were similar in the two groups (Table 2).

**Table 1:** General hemodynamics and ventilation over time in the two groups of animals

	T-1	T0	T3	T6	T9	T12	T15	T18
HR, beats/min								
Control	98 ± 12	95 ± 12	108 ± 12	108 ± 16	110 ± 9*	108 ± 14	116 ± 24	111 ± 38
Denervation	96 ± 10	109 ± 10	112 ± 24*	113 ± 23*	102 ± 19	112 ± 25	117 ± 30	106 ± 35
CI, L/min/m^2^								
Control	5.3 ± 1.1	5.2 ± 1.4	5.4 ± 1.2	5.4 ± 1.3	5.4 ± 1.6	4.5 ± 1.4	4.5 ± 1.9	3.7 ± 1.8
Denervation	4.8 ± 1.0	6.5 ± 1.2*	5.9 ± 1.9	6.1 ± 1.2	4.9 ± 1.2	4.5 ± 1.5	3.8 ± 1.6	3.8 ± 2.3
MAP, mmHg								
Control	88 ± 11	85 ± 7	81 ± 13	73 ± 21	59 ± 19*	43 ± 8*	39 ± 7*	33 ± 7*
Denervation	90 ± 10	85 ± 6	86 ± 12	78 ± 9*	62 ± 18*	48 ± 9*	43 ± 11*	41 ± 13*
SVRI, dynes·s/cm^5^/m^2^								
Control	1300 ± 270	1308 ± 290	1236 ± 448	1063 ± 340*	828 ± 204*	720 ± 181*	659 ± 172*	640 ± 225*
Denervation	1460 ± 198	1028 ± 199*	1187 ± 362	990 ± 136*	920 ± 186*	843 ± 217*	889 ± 349*	950 ± 685
SvO_2_, %								
Control	73.6 ± 4.4	74.3 ± 4.8	75.8 ± 8.0	77.8 ± 7.0	74.4 ± 9.3	70.8 ± 9.7	68.9 ± 8.0	54.3 ± 10.0
Denervation	76.0 ± 6.0	80.9 ± 2.9	78.4 ± 3.6	82.5 ± 3.0	77.9 ± 2.9	73.3 ± 4.8	64.8 ± 14.5	56.1 ± 18.9
Fluid administered, mL								
Control	536 ± 78	978 ± 186*	2238 ± 239*	3610 ± 336*	4790 ± 399*	5816 ± 457*	6662 ± 470*	7506 ± 469*
Denervation	632 ± 87	1328 ± 278*	2650 ± 375*	3800 ± 516*	4630 ± 533*	5528 ± 486*	6336 ± 486*	7328 ± 442*
PAOP, mmHg								
Control	4 ± 2	4 ± 2	4 ± 2	5 ± 2	4 ± 2	5 ± 3	5 ± 4	7 ± 4
Denervation	5 ± 2	4 ± 2	4 ± 2	4 ± 3	5 ± 3	5 ± 2	5 ± 2	6 ± 3
P/F								
Control	477 ± 50	478 ± 70	442 ± 111	427 ± 148	395 ± 177	366 ± 180	313 ± 175*	187 ± 146*
Denervation	467 ± 47	457 ± 63	426 ± 71	435 ± 53*	384 ± 76*	318 ± 108*	265 ± 119*	231 ± 126*

*HR* heart rate, *CI* cardiac index, *MAP* mean arterial pressure, *SVRI* systemic vascular resistance index, *SvO*_*2*_ mixed venous oxygen saturation, *PAOP* pulmonary artery balloon-occluded pressure

*p < 0.05 vs. baseline

**Table 2:** Time to event analyses in the two groups of animals

	Control, h	Denervation, h	p (Log Rank)
Shock (hours until MAP <65 mmHg)	8 [3–11]	9 [7–11]	0.805
Renal hypoperfusion (hours until 0.5 × baseline)	11 [4–18]	10 [9–18]	0.759
LPR_cortex_ (hours until >32.7)	16 [10–18]	14 [8–18]	0.664
Oliguria (hours until UO < 0.5 mL/kg/h)	11 [6–18]	10 [6–12]	0.964
Plasma creatinine (hours until 1.5 × baseline)	14 [9–18]	12 [9–15]	0.492
Creatinine clearance (hours until 0.5 × baseline)	12 [6–12]	11 [6–12]	0.665
Survival (hours from baseline)	21 [18–24]	21 [18–25]	0.496

Data are presented as median [range]

*MAP* mean arterial pressure, *LPR*_*cortex*_ cortical lactate/pyruvate ratio, *UO* urine output

**Table 3:** Systemic metabolism over time in the two groups of animals

	T-1	T0	T3	T6	T9	T12	T15	T18
DO_2_I, L/min/m^2^								
Control	645 ± 162	598 ± 102	685 ± 157	687 ± 143	688 ± 223	578 ± 199	537 ± 175	366 ± 122*
Denervation	617 ± 133	763 ± 121*	749 ± 238	759 ± 184*	677 ± 165	597 ± 187	486 ± 23	458 ± 390
VO_2_I, L/min/m^2^								
Control	177 ± 38	164 ± 33	167 ± 49	155 ± 36	162 ± 37	147 ± 43	137 ± 25*	129 ± 55
Denervation	148 ± 19	156 ± 9	157 ± 34	139 ± 48	147 ± 37	157 ± 49	141 ± 48	142 ± 68
O_2_ER, %								
Control	28 ± 5	28 ± 4	25 ± 9	23 ± 8	24 ± 6	26 ± 6	28 ± 8	35 ± 9
Denervation	25 ± 6	21 ± 3	22 ± 2	18 ± 3*	22 ± 4	27 ± 4	32 ± 11	38 ± 12
Hb, g/dL								
Control	8.6 ± 0.6	8.4 ± 1.0	9.2 ± 1.1	9.2 ± 0.9	9.5 ± 0.6*	9.7 ± 0.4*	9.4 ± 0.9*	9.3 ± 1.2
Denervation	9.3 ± 1.0	8.6 ± 0.8*	9.2 ± 1.2	9.1 ± 1.5	10.2 ± 1.7	10.1 ± 1.7	9.6 ± 1.5	9.2 ± 1.5
Epinephrine, μg/L								
Control	-	0.18 ± 0.08	-	0.26 ± 0.06	-	1.05 ± 0.63*	-	3.10 ± 2.25*
Denervation	-	0.22 ± 0.16	-	0.50 ± 0.39	-	1.22 ± 0.58*	-	2.33 ± 1.39*
Glucose, mg/dL								
Control	60 ± 10	57 ± 13	56 ± 18	47 ± 6*	45 ± 11	45 ± 11	52 ± 11	48 ± 8*
Denervation	58 ± 10	54 ± 6	52 ± 14	53 ± 9	51 ± 14	47 ± 11	51 ± 11	52 ± 24
Lactate, mmol/L								
Control	1.1 ± 0.3	1.0 ± 0.3	1.1 ± 0.5	1.2 ± 0.6	1.9 ± 0.9*	3.5 ± 2.1*	6.2 ± 3.6*	10.8 ± 3.9*
Denervation	1.2 ± 0.3	1.5 ± 0.4	1.3 ± 0.9	1.5 ± 0.8	2.3 ± 0.8*	2.9 ± 1.1*	4.5 ± 1.4*	8.1 ± 3.5*
Pyruvate, μmol/L								
Control	43 ± 8	36 ± 5	54 ± 19	68 ± 19*	92 ± 15*	148 ± 22*	202 ± 43*	208 ± 26*
Denervation	47 ± 11	55 ± 24	55 ± 21	83 ± 26*	102 ± 33*	127 ± 21*	155 ± 29*	224 ± 50*
P_v-a_CO_2_, mmHg								
Control	4.7 ± 2.4	4.9 ± 0.9	4.8 ± 1.9	5.0 ± 1.7	5.6 ± 2.5	7.2 ± 3.3	7.5 ± 3.2	12.9 ± 7.0*
Denervation	5.7 ± 1.0	3.5 ± 1.5*	4.8 ± 1.9	4.7 ± 1.5	5.7 ± 1.8	6.1 ± 2.1	9.7 ± 4.2	10.5 ± 7.2

*DO*_*2*_ *I* oxygen delivery index, *VO*_*2*_ *I* oxygen consumption index, *O*_*2*_ *ER* oxygen extraction ratio, *Hb* hemoglobin, *P*_*v-a*_ *CO*_*2*_ veno-arterial carbon dioxide tension difference

*p < 0.05 vs. baseline

### Renal hemodynamics and metabolism

**Fig. 2:**
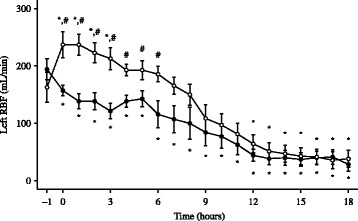
Evolution of left renal blood flow (RBF) in denervated (*n* = 7, *open circles*) and control (*n* = 7, filled circles) animals. Data are presented as mean ± SEM. *p < 0.05 vs. baseline, #*p* < 0.05 vs. control

**Fig. 3:**
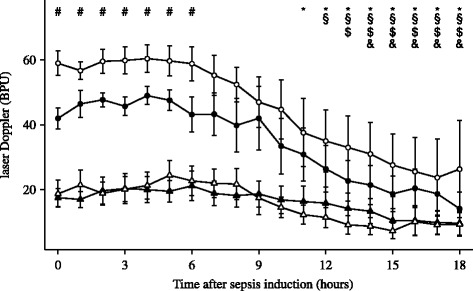
Changes in cortical (*circles*) and medullary (*triangles*) laser Doppler values in denervated (*n* = 7, open symbols) and control (*n* = 7, filled symbols) animals. Data are presented as mean ± SEM. #*p* < 0.05 cortical laser Doppler denervated vs. control., *p < 0.05 cortical laser Doppler vs. baseline in denervated animals, §*p* < 0.05 cortical laser Doppler vs. baseline in control animals, $*p* < 0.05 medullary laser Doppler vs. baseline in denervated animals, &*p* < 0.05 medullary laser Doppler vs. baseline in control animals. To avoid the risk of traumatic injury, probes were inserted after surgical denervation

**Fig. 4:**
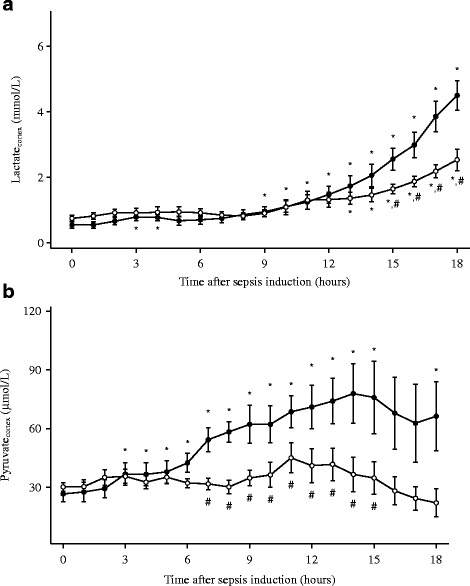
Changes in interstitial lactate (**a**) and pyruvate (**b**) in the renal cortex of denervated (*n* = 7, *open circles*) and control (*n* = 7, *filled circles*) animals. Data are presented as mean ± SEM. *p < 0.05 vs. baseline, #*p* < 0.05 vs. control

Absolute and relative RBF increased immediately after the denervation procedure and both were higher at T0 in the denervated than in the non-denervated animals. RBF in the control group was decreased at T0 compared to T-1 (Fig. 2). Cortical perfusion and RBF were higher in denervated than in non-denervated animals until 6 h after the induction of sepsis while medullary perfusion decreased similarly in both groups (Fig. 3). Renal VO_2_ decreased over time in both groups (Table 4). Cortical pyruvate and lactate increased significantly in the control group from 3 and 9 h, respectively, after induction of sepsis (Fig. 4). Cortical pyruvate in the denervated animals remained unchanged while cortical lactate increased after 13 h of sepsis. Cortical lactate was lower in the denervated animals than in the controls from 15 h after sepsis (*p* < 0.001). ΔPyruvate_cortex_ was different between the groups from T6, suggesting that the interstitial changes largely reflected local alterations (Table 4). ΔLactate_cortex_ increased similarly in both groups.

**Table 4:** Renal hemodynamics and metabolism over time in the two groups of animals

	T-1	T0	T3	T6	T9	T12	T15	T18
RBF, mL/min								
Control	194 ± 48	157 ± 24*	121 ± 36*	116 ± 62*	84 ± 61*	44 ± 28*	37 ± 30*	31 ± 29*
Denervation	163 ± 69	237 ± 59*,^#^	213 ± 50*,^#^	186 ± 36^#^	109 ± 64	64 ± 42*	43 ± 39*	38 ± 35*
RBF/CO, %								
Control	4.8 ± 0.2	4.1 ± 33	2.9 ± 0.8*	2.6 ± 0.9*	1.8 ± 0.1*	1.2 ± 0.5*	0.9 ± 0.5*	0.8 ± 0.7*
Denervation	4.5 ± 0.2	4.9 ± 9	5.0 ± 1.5^#^	4.1 ± 0.9^#^	2.8 ± 1.7	1.9 ± 0.8*	1.2 ± 0.9*	1.3 ± 0.1*
Renal DO_2_I, mL/min/m^2^								
Control	-	14.3 ± 4	12.2 ± 4.1	11.6 ± 6.7	8.2 ± 6.8	4.1 ± 2.9*	4.0 ± 2.4*	2.7 ± 1.8*
Denervation	-	21.6 ± 3	21.1 ± 7.8^#^	17.8 ± 5.3	10.9 ± 5.7*	6.3 ± 4.0*	4.2 ± 3.8*	3.5 ± 3.4*
Renal VO_2_I, mL/min/m^2^								
Control	-	2.0 ± 1.0	1.7 ± 0.6	1.3 ± 0.5	1.1 ± 0.5*	0.9 ± 0.5*	0.9 ± 0.2*	0.9 ± 0.3*
Denervation	-	1.9 ± 0.8	1.9 ± 0.5	1.4 ± 0.4	1.0 ± 0.4*	1.2 ± 0.6	0.6 ± 0.3*	0.7 ± 0.6*
Renal O_2_ER, %								
Control	-	14 ± 4	15 ± 5	13 ± 5	20 ± 11	32 ± 19	33 ± 21	45 ± 23
Denervation	-	10 ± 6	10 ± 3	8 ± 3	11 ± 6	21 ± 10	32 ± 24	31 ± 16
Laser Doppler_cortex_, BPU								
Control	-	42 ± 9	46 ± 7	43 ± 14	42 ± 26	26 ± 19*	19 ± 15*	14 ± 13*
Denervation	-	59 ± 10^#^	60 ± 11^#^	59 ± 14^#^	47 ± 21	35 ± 25	28 ± 26*	26 ± 17
Laser Doppler_medulla_, BPU								
Control	-	18 ± 7	20 ± 11.0	21 ± 11	19 ± 10	16 ± 12	11 ± 9*	10 ± 9*
Denervation	-	19 ± 11	20 ± 10	23 ± 12	17 ± 14	12 ± 8	7 ± 6*	9 ± 8*
Lactate_cortex_, mmol/L								
Control	-	0.5 ± 0.3	0.8 ± 0.3*	0.7 ± 0.3	0.9 ± 0.4*	1.5 ± 0.7*	2.6 ± 0.9*	4.5 ± 1.2*
Denervation	-	0.7 ± 0.2	0.9 ± 0.4	0.9 ± 0.3	0.9 ± 0.3	1.3 ± 0.6	1.6 ± 0.4*,^#^	2.5 ± 0.8*,^#^
ΔLactate_cortex_, mmol/L								
Control	-	−0.3 ± 0.3	−0.3 ± 0.3	−0.5 ± 0.3	−1.0 ± 0.6*	−2.1 ± 1.6*	−3.8 ± 3.1*	−6.6 ± 3.3*
Denervation	-	−0.8 ± 0.3	−0.3 ± 0.5	−0.5 ± 0.5	−1.3 ± 0.7	−1.4 ± 1.1	−2.8 ± 1.5*	−5.6 ± 3.4*
Pyruvate_cortex_, mmol/L								
Control	-	27 ± 11	41 ± 23*	42 ± 13*	62 ± 26*	75 ± 29*	76 ± 49*	66 ± 46.0
Denervation	-	30 ± 6	36 ± 10	32 ± 6	35 ± 11^#^	41 ± 23^#^	35 ± 22	22 ± 17^#^
ΔPyruvate_cortex_, mmol/L								
Control	-	−10 ± 10	−17 ± 10	−27 ± 21*	−41 ± 13*	−91 ± 38*	−150 ± 51*	−150 ± 51*
Denervation	-	−26 ± 21	−18 ± 11	−50 ± 17*,^#^	−67 ± 16*,^#^	−82 ± 27*	−115 ± 22*	−200 ± 32*,^#^
LPR_cortex_, mmol/L								
Control	-	19.3 ± 6	21.9 ± 6.7	17.5 ± 8.3	17 ± 6.8	22.7 ± 161.1	51.2 ± 43.7	137.7 ± 126.2
Denervation	-	26.3 ± 5.7	25.7 ± 6.2	29.2 ± 9.8	28.5 ± 12.5	43 ± 36.4	74.8 ± 52.9*	177.9 ± 106.3*
Glucose_cortex_, mg/dL								
Control	-	21 ± 8	18 ± 7	22 ± 12	25 ± 9	22 ± 13	21 ± 15	18 ± 17
Denervation	-	26 ± 5	25 ± 7	25 ± 6	23 ± 8	25 ± 10	22 ± 13	12 ± 11*

*RBF* renal blood flow, *RBF/CO* relative renal blood flow, *DO*_*2*_ *I* oxygen delivery index, *VO*_*2*_ *I* oxygen consumption index, *O*_*2*_ *ER* oxygen extraction ratio, *ΔLactate*_*cortex*_ plasma to cortical lactate gradient, *ΔPyruvate*_*cortex*_ plasma to cortical pyruvate gradient, *LPR*_*cortex*_ cortical lactate/pyruvate ratio

*p < 0.05 vs. baseline, ^#^ *p* < 0.05 vs. control

### Renal function

Creatinine clearance decreased similarly in both groups (Table 5). Time to oliguria, defined as UO < 0.5 mL/kg/h, and time to increased plasma creatinine, defined as 1.5 times baseline, were similar in the denervated and control groups (Table 2).

**Table 5:** Renal function over time in the two groups of animals

	T-1	T0	T3	T6	T9	T12	T15	T18
UO, mL/h								
Control	57 ± 31	62 ± 36	71 ± 41	51 ± 16	39 ± 66	3 ± 4*	2 ± 4*	1 ± 2*
Denervation	66 ± 47	84 ± 70	84 ± 46	53 ± 23*	37 ± 20	10 ± 10*	1 ± 3*	0 ± 0*
Fluid balance, mL/h								
Control	211 ± 79	369 ± 168*	749 ± 145*	1256 ± 180*	1723 ± 195*	2209 ± 284*	2625 ± 304*	3043 ± 311*
Denervation	260 ± 114	625 ± 221*,^#^	912 ± 286*	1315 ± 355*	1600 ± 366*	2003 ± 355*	2394 ± 365*	2860 ± 386*
Creatinine clearance, mL/min								
Control	61 ± 17	61 ± 18	80 ± 42	59 ± 42	45 ± 51	5 ± 6*	2.0 ± 0.4*	1 ± 2*
Denervation	77 ± 43	54 ± 29	62 ± 27	58 ± 32	42 ± 30	7 ± 8*	1.9 ± 0.6*	0 ± 0*
Plasma creatinine, mg/dL								
Control	0.9 ± 0.1	0.9 ± 0.1	0.8 ± 0.1*	0.8 ± 0.1	1.0 ± 0.4	1.6 ± 0.4*	2.0 ± 0.4*	2.5 ± 0.5*
Denervation	0.7 ± 0.2	0.8 ± 0.2	0.8 ± 0.2	0.7 ± 0.2	0.9 ± 0.3	1.4 ± 0.5*	1.9 ± 0.6*	2.3 ± 0.9*
FF, %								
Control	21 ± 8	22 ± 7	32 ± 14	25 ± 14	21 ± 19	4 ± 4*	1 ± 2*	0 ± 1*
Denervation	27 ± 10	15 ± 6*	20 ± 10	20 ± 9	27 ± 24	8 ± 7	1 ± 1*	0 ± 0*
TNa^+^, mmol/min								
Control	8.4 ± 2.4	8.4 ± 2.6	11.4 ± 6.4	9.0 ± 5.4	7.2 ± 7.0	0.8 ± 0.8*	0.3 ± 0.6*	0.1 ± 0.2*
Denervation	10.5 ± 4.2	7.2 ± 4.1	8.2 ± 3.6	7.8 ± 4.3	5.7 ± 4.3	1.0 ± 1.1*	0.1 ± 0.2*	0.0 ± 0.0*
FENa^+^, %								
Control	1.4 ± 1.0	1.3 ± 0.5	1.3 ± 0.5	1.0 ± 0.6	0.8 ± 0.7	0.4 ± 0.5	0.0 ± 0.0*	0.0 ± 0.0*
Denervation	2.2 ± 1.6	1.9 ± 1.5	2.2 ± 1.6	1.4 ± 0.8	1.3 ± 1.1	0.9 ± 1.2	0.0 ± 0.0*	0.0 ± 0.0*

*UO* urine output, *FF* filtration fraction, *TNa* ^+^ sodium reabsorption, *FENa* ^+^ fractional excretion of sodium

*p < 0.05 vs. baseline, ^#^ *p* < 0.05 vs. control

## Discussion

Renal denervation effectively increased RBF and cortical perfusion during early, normotensive sepsis whereas medullary flow was unaffected. The increase in RBF was transient and, after hypotension, values in denervated animals decreased to the same levels as in controls. Cortical pyruvate increased during early sepsis in the non-denervated animals but remained unchanged in the denervated group. The late increase in cortical lactate seemed to be attenuated in the denervated animals, although this may also reflect lower systemic values. Approximately 13 h after induction of sepsis, the cortical L/P ratio increased rapidly in both groups, reflecting the development of tissue hypoxia [31, 32]. This increase appeared more pronounced in the denervated group. There were no differences in the evolutions of plasma creatinine, creatinine clearance, urine output or in time to oliguria. There were no differences in survival between the groups.

Findings on the impact of kidney perfusion on renal function in sepsis are contradictory. For example, Johannes et al. reported that renal dysfunction with hypoperfusion was more severe in endotoxemic rats than in flow-matched healthy controls [33]. In an ovine model of infused live bacteria, Langenberg et al. reported an increased RBF associated with a decrease in creatinine clearance [9]. In at least one study on renal perfusion in human septic AKI, the absolute value of RBF, assessed with phase-contrast magnetic resonance imaging, was increased compared to healthy controls [11]. However, a consistent decrease in relative RBF, i.e., the fraction of cardiac output directed to the kidneys, was also observed [10, 11]. This suggests that there may still be substantial perfusion-metabolism mismatch that warrants therapeutic intervention. Indeed, in two models of porcine sepsis, Benes et al. showed that this flow redistribution separated the animals with and without renal dysfunction [34]. The selective decrease in RBF may be the result of several factors, including the intrinsic properties of renal autoregulation, the release of intrarenal vasoconstrictors and possibly increased sympathetic input [35–38].

Attempts to selectively increase RBF have not always been successful [13, 14]. We recently reported that the administration of fenoldopam in our ovine model of experimental septic shock failed to preserve RBF or improve renal function [39]. In the present study, acute denervation caused an increase in cortical perfusion and a doubling of RBF in the early phase of sepsis. It also reduced cortical pyruvate levels, suggesting an attenuation of glycolytic activity in this phase [40].

Hyperlactatemia in septic shock may be caused primarily by tissue hypoperfusion and hypoxia, but adrenergic induction of aerobic glycolysis by circulating epinephrine, through β_2_ stimulation, likely also contributes [41, 42]. Renal denervation, however, did not alter plasma epinephrine levels and it appears that local tissue metabolism was affected by the change in RBF, possibly through a reduction in renal vascular resistance that was evident during the first few hours [43–45].

Although renal denervation augmented cortical perfusion, it did not affect medullary flow at any stage. The renal medulla is sparsely innervated and so any effect of reduced RSNA was unlikely to benefit this part of the kidney, which is thought to be in a continuous state of near-hypoxia [20, 46]. A recent study in ovine sepsis suggested that medullary ischemia may be implicated in the development of septic AKI, and the intrarenal redistribution of blood flow that followed denervation could explain the absence of any beneficial effect on renal function [47].

The effect of denervation on RBF and cortical perfusion disappeared after the onset of shock, and was followed by a reduction in renal VO_2_ and an elevated cortical L/P ratio. It therefore seems that the flow redistribution during septic shock, which occurred in the absence of any renal sympathetic input, still resulted in a relevant perfusion-metabolism mismatch. Moreover, the increase in the cortical L/P ratio in this phase seemed more pronounced in the denervated animals, suggesting that flow was less efficiently matched to metabolism. This, and the associated trend to a lower renal O_2_ER, is in line with experiments showing that alpha blockade is associated with impaired oxygen extraction and earlier onset of tissue hypoxia [48, 49].

Several limitations might complicate the interpretation of our findings.

First, using HES 6% as a resuscitation fluid may have negatively affected renal function in our model [50]. However, a certain amount of colloid administration is necessary to limit edema formation, and human albumin could not be given. Moreover, the total amount of HES given was limited and we believe HES will have had only a minor, if any, effect on renal function in our model.

Second, fluid resuscitation was initiated immediately following the induction of sepsis, preventing us from observing the effects of early renal hypoperfusion. Given that early oxygenation deficits have been proposed to be a reflection of an upstream perfusion deficit [51], augmenting renal perfusion in non-resuscitated sepsis could have resulted in a different response than the one we observed.

Third, the microdialysis technique does not allow direct observation of the mechanisms underlying changes in interstitial metabolite concentrations. For example, increased renal oxygenation of lactate or gluconeogenesis could also explain lower pyruvate and lactate concentrations [52–54]. Alternatively, increased pyruvate dehydrogenase (PDH) activity may have contributed to lower lactate and pyruvate levels during early sepsis [55–57]. This would, however, be in contrast with studies that have shown an increased PDH activity by norepinephrine in white and brown adipose tissue in vivo [58] and in isolated hepatocytes and myocardial cells [59, 60]. Moreover, norepinephrine induces systemic gluconeogenesis and a renal effect in the same direction by denervation seems unlikely [61, 62]. Similarly, mitochondrial dysfunction could have contributed to the higher cortical L/P ratios that were observed in the denervated animals during septic shock [63].

Fourth, as we wanted to study the effects of denervation in the absence of pharmacological interference, we did not administer any vasopressor agents. We therefore cannot directly translate our data to situations in which MAP would be corrected. Similar reasoning applies to other supportive measures, such as source control or the administration of antibiotics.

Fifth, application of acute renal denervation in a less lethal model may have had different, possibly even beneficial, effects on kidney function. However, Calzavacca et al. [19] observed no beneficial effect on renal function when applying chronic denervation in a less severe model of septic AKI. Hence, a beneficial effect of acute denervation in the same model seems unlikely.

Finally, given that the perfusion-metabolism mismatch occurred after the hemodynamic effect of denervation had disappeared, our findings fail to provide an answer to the question regarding whether targeting renal perfusion is still beneficial in this more relevant phase.

## Conclusions

Acute renal denervation increased RBF and cortical perfusion and attenuated metabolic changes in the renal cortex during early, normotensive sepsis. Medullary perfusion was unaffected, possibly resulting in an intrarenal maldistribution of RBF. Denervation failed to prevent renal hypoperfusion and the associated perfusion-metabolism mismatch that occurred after shock. Renal denervation did not affect kidney function or survival.

## Abbreviations

AKI: Acute kidney injury
BPU: Blood perfusion units
CI: Cardiac index
DO_2_I: Oxygen delivery index
FENa: Fractional excretion of sodium
FF: Filtration fraction
HES: Hydroxyethyl starch
L/P: Lactate/pyruvate ratio
MAP: Mean arterial pressure
O_2_ER: Oxygen extraction ratio
P/F ratio: Ratio of arterial oxygen partial pressure to fractional inspired oxygen
PAOP: Pulmonary artery balloon-occluded pressure
PDH: Pyruvate dehydrogenase
RBF: Renal blood flow
RSNA: Renal sympathetic nerve activity
SVRI: Systemic vascular resistance index
TNa^+^: Sodium reabsorption
TNa^+^: Sodium reabsorption
UO: Urine output
VO_2_: Oxygen consumption
VO_2_I: Oxygen consumption index
ΔLactate_cortex_: Plasma-interstitium pyruvate gradient in the renal cortex
ΔPyruvate_cortex_: Plasma-interstitium pyruvate gradient in the renal cortex

## References

[1] Uchino S, Kellum JA, Bellomo R, Doig GS, Morimatsu H, Morgera S (2005). Acute renal failure in critically ill patients: a multinational, multicenter study. JAMA.

[2] Bagshaw SM, George C, Bellomo R (2008). ANZICS database management committee. Early acute kidney injury and sepsis: a multicentre evaluation. Crit Care.

[3] Sakhuja A, Kumar G, Gupta S, Mittal T, Taneja A, Nanchal RS (2015). Acute kidney injury requiring dialysis in severe sepsis. Am J Respir Crit Care Med.

[4] Oppert M, Engel C, Brunkhorst F-M, Bogatsch H, Reinhart K, Frei U (2008). Acute renal failure in patients with severe sepsis and septic shock–a significant independent risk factor for mortality: results from the German prevalence study. Nephrol Dial Transplant.

[5] Wu L, Gokden N, Mayeux PR (2007). Evidence for the role of reactive nitrogen species in polymicrobial sepsis-induced renal peritubular capillary dysfunction and tubular injury. J Am Soc Nephrol.

[6] Chvojka J, Sykora R, Krouzecky A, Radej J, Varnerova V, Karvunidis T (2008). Renal haemodynamic, microcirculatory, metabolic and histopathological responses to peritonitis-induced septic shock in pigs. Crit Care.

[7] Seely KA, Holthoff JH, Burns ST, Wang Z, Thakali KM, Gokden N (2011). Hemodynamic changes in the kidney in a pediatric rat model of sepsis-induced acute kidney injury. Am J Physiol Ren Physiol.

[8] Post EH, Kellum JA, Bellomo R, Vincent JL (2017). Renal perfusion in sepsis: from macro- to microcirculation. Kidney Int.

[9] Langenberg C, Wan L, Egi M, May CN, Bellomo R (2006). Renal blood flow in experimental septic acute renal failure. Kidney Int.

[10] Brenner M, Schaer GL, Mallory DL, Suffredini AF, Parrillo JE (1990). Detection of renal blood flow abnormalities in septic and critically ill patients using a newly designed indwelling thermodilution renal vein catheter. Chest.

[11] Prowle JR, Molan MP, Hornsey E, Bellomo R (2012). Measurement of renal blood flow by phase-contrast magnetic resonance imaging during septic acute kidney injury: a pilot investigation. Crit Care Med.

[12] Post EH, Su F, Hosokawa K, Taccone FS, Herpain A, Creteur J (2017). Changes in kidney perfusion and renal cortex metabolism in septic shock: an experimental study. J Surg Res.

[13] Day NP, Phu NH, Mai NT, Bethell DB, Chau TT, Loc PP (2000). Effects of dopamine and epinephrine infusions on renal hemodynamics in severe malaria and severe sepsis. Crit Care Med.

[14] Lauschke A, Teichgräber UKM, Frei U, Eckardt KU (2006). “low-dose”dopamine worsens renal perfusion in patients with acute renal failure. Kidney Int.

[15] Schlaich MP, Lambert E, Kaye DM, Krozowski Z, Campbell DJ, Lambert G (2004). Sympathetic augmentation in hypertension: role of nerve firing, norepinephrine reuptake, and angiotensin neuromodulation. Hypertension.

[16] Malpas SC (2010). Sympathetic nervous system overactivity and its role in the development of cardiovascular disease. Physiol Rev.

[17] Ramchandra R, Wan L, Hood SG, Frithiof R, Bellomo R, May CN. Septic shock induces distinct changes in sympathetic nerve activity to the heart and kidney in conscious sheep. Am J Physiol Regul Integr Comp Physiol. 2009;297:R1247–53. 10.1152/ajpregu.00437.2009 19726712

[18] Wang W, Falk SA, Jittikanont S, Gengaro PE, Edelstein CL, Schrier RW (2002). Protective effect of renal denervation on normotensive endotoxemia-induced acute renal failure in mice. Am J Physiol Ren Physiol.

[19] Calzavacca P, Bailey M, Velkoska E, Burrell LM, Ramchandra R, Bellomo R (2014). Effects of renal denervation on regional hemodynamics and kidney function in experimental hyperdynamic sepsis. Crit Care Med.

[20] Kompanowska-Jezierska E, Walkowska A, Johns EJ, Sadowski J (2001). Early effects of renal denervation in the anaesthetised rat: natriuresis and increased cortical blood flow. J Physiol.

[21] Tsioufis C, Papademetriou V, Dimitriadis K, Tsiachris D, Thomopoulos C, Park E (2013). Catheter-based renal sympathetic denervation exerts acute and chronic effects on renal hemodynamics in swine. Int J Cardiol.

[22] McGrath JC, Drummond GB, McLachlan EM, Kilkenny C, Wainwright CL (2010). Guidelines for reporting experiments involving animals: the ARRIVE guidelines. Br J Pharmacol.

[23] Linz D, Wirth K, Ukena C, Mahfoud F, Pöss J, Linz B (2013). Renal denervation suppresses ventricular arrhythmias during acute ventricular ischemia in pigs. Heart Rhythm.

[24] Taccone FS, Su F, De Deyne C, Abdellhai A, Pierrakos C, He X (2014). Sepsis is associated with altered cerebral microcirculation and tissue hypoxia in experimental peritonitis. Crit Care Med.

[25] Salgado DR, He X, Su F, de Sousa DB, Penaccini L, Maciel LK (2011). Sublingual microcirculatory effects of enalaprilat in an ovine model of septic shock. Shock.

[26] Singer M, Deutschman CS, Seymour CW, Shankar-Hari M, Annane D, Bauer M (2016). The third international consensus definitions for sepsis and septic shock (sepsis-3). JAMA.

[27] Souba W (2001). Surgical research.

[28] Hansen DK, Davies MI, Lunte SM, Lunte CE (1999). Pharmacokinetic and metabolism studies using microdialysis sampling. J Pharm Sci.

[29] Gerlo E, Malfait R (1985). High-performance liquid chromatographic assay of free norepinephrine, epinephrine, dopamine, vanillylmandelic acid and homovanillic acid. J Chromatogr.

[30] Lloyd B, Burrin J, Smythe P, Alberti KG (1978). Enzymic fluorometric continuous-flow assays for blood glucose, lactate, pyruvate, alanine, glycerol, and 3-hydroxybutyrate. Clin Chem.

[31] Weil MH, Afifi AA (1970). Experimental and clinical studies on lactate and pyruvate as indicators of the severity of acute circulatory failure (shock). Circulation.

[32] Levy B, Sadoune LO, Gelot AM, Bollaert P-E, Nabet P, Larcan A (2000). Evolution of lactate/pyruvate and arterial ketone body ratios in the early course of catecholamine-treated septic shock. Crit Care Med.

[33] Johannes T, Mik EG, Ince C (2009). Nonresuscitated endotoxemia induces microcirculatory hypoxic areas in the renal cortex in the rat. Shock.

[34] Benes J, Chvojka J, Sykora R, Radej J, Krouzecky A, Novak I (2011). Searching for mechanisms that matter in early septic acute kidney injury: an experimental study. Crit Care.

[35] Badr KF (1992). Sepsis-associated renal vasoconstriction: potential targets for future therapy. Am J Kidney Dis.

[36] Boffa JJ, Arendshorst WJ (2005). Maintenance of renal vascular reactivity contributes to acute renal failure during endotoxemic shock. J Am Soc Nephrol.

[37] Henrich WL, Hamasaki Y, Said SI, Campbell WB, Cronin RE (1982). Dissociation of systemic and renal effects in endotoxemia. Prostaglandin inhibition uncovers an important role of renal nerves. J Clin Invest.

[38] Fantini GA, Shiono S, Bal BS, Shires GT (1989). Adrenergic mechanisms contribute to alterations in regional perfusion during normotensive E. coli bacteremia. J Trauma.

[39] Post EH, Su F, Taccone FS, Hosokawa K, Herpain A, Creteur J (2016). The effects of fenoldopam on renal function and metabolism in an ovine model of septic shock. Shock.

[40] Levy B (2006). Lactate and shock state: the metabolic view. Curr Opin Crit Care.

[41] James JH, Fang CH, Schrantz SJ, Hasselgren PO, Paul RJ, Fischer JE (1996). Linkage of aerobic glycolysis to sodium-potassium transport in rat skeletal muscle. Implications for increased muscle lactate production in sepsis. J Clin Invest.

[42] James JH, Luchette FA, McCarter FD, Fischer JE (1999). Lactate is an unreliable indicator of tissue hypoxia in injury or sepsis. Lancet.

[43] Johns EJ, Kopp UC, DiBona GF (2011). Neural control of renal function. Compr Physiol.

[44] Johannes T, Mik EG, Nohé B, Raat NJH, Unertl KE, Ince C (2006). Influence of fluid resuscitation on renal microvascular PO2 in a normotensive rat model of endotoxemia. Crit Care.

[45] Redfors B, Bragadottir G, Sellgren J, Swärd K, Ricksten SE (2011). Effects of norepinephrine on renal perfusion, filtration and oxygenation in vasodilatory shock and acute kidney injury. Intensive Care Med.

[46] Evans RG, Gardiner BS, Smith DW, O’Connor PM (2008). Intrarenal oxygenation: unique challenges and the biophysical basis of homeostasis. Am J Physiol Ren Physiol.

[47] Calzavacca P, Evans RG, Bailey M, Bellomo R, May CN. Cortical and medullary tissue perfusion and oxygenation in experimental septic acute kidney injury. Crit Care Med. 2015;43:e431-9 10.1097/CCM.0000000000001198 26181218

[48] Cain SM (1978). Effects of time and vasoconstrictor tone on O2 extraction during hypoxic hypoxia. J Appl Physiol.

[49] Maginniss LA, Connolly H, Samsel RW, Schumacker PT (1994). Adrenergic vasoconstriction augments tissue O2 extraction during reductions in O2 delivery. J Appl Physiol.

[50] Perner A, Haase N, Guttormsen AB, Tenhunen J, Klemenzson G, Åneman A (2012). Hydroxyethyl starch 130/0.42 versus Ringer’s acetate in severe sepsis. N. Engl J Med.

[51] Wang Z, Sims CR, Patil NK, Gokden N, Mayeux PR (2014). Pharmacologic targeting of sphingosine-1-phosphate receptor 1 improves the renal microcirculation during sepsis in the mouse. J Pharmacol Exp Ther.

[52] Albuszies G, Vogt J, Wachter U, Thiemermann C, Leverve XM, Weber S (2007). The effect of iNOS deletion on hepatic gluconeogenesis in hyperdynamic murine septic shock. Intensive Care Med.

[53] Simkova V, Baumgart K, Vogt J, Wachter U, Weber S, Gröger M (2008). The effect of superoxide dismutase overexpression on hepatic gluconeogenesis and whole-body glucose oxidation during resuscitated normotensive murine septic shock. Shock.

[54] Kelmer-Bracht AM, Broetto-Biazon AC, Sá-Nakanishi D, Babeto A, Ishii-Iwamoto EL, Bracht A (2006). Low doses of tumour necrosis factor α and interleukin 1β diminish hepatic gluconeogenesis from alanine in vivo. Basic Clin Pharmacol Toxicol.

[55] Stacpoole PW, Wright EC, Baumgartner TG, Bersin RM, Buchalter S, Curry SH (1992). A controlled clinical trial of dichloroacetate for treatment of lactic acidosis in adults. The Dichloroacetate-lactic acidosis study group. N Engl J Med.

[56] Curtis SE, Cain SM (1992). Regional and systemic oxygen delivery/uptake relations and lactate flux in hyperdynamic, endotoxin-treated dogs. Am Rev Respir Dis.

[57] Vary TC (1996). Sepsis-induced alterations in pyruvate dehydrogenase complex activity in rat skeletal muscle: effects on plasma lactate. Shock.

[58] Trayhurn P, Ashwell M (1987). Control of white and brown adipose tissues by the autonomic nervous system. Proc Nutr Soc.

[59] Assimacopoulos-Jeannet F, McCormack JG, Jeanrenaud B (1983). Effect of phenylephrine on pyruvate dehydrogenase activity in rat hepatocytes and its interaction with insulin and glucagon. FEBS Lett.

[60] Di Lisa F, Fan CZ, Gambassi G, Hogue BA, Kudryashova I, Hansford RG. Altered pyruvate dehydrogenase control and mitochondrial free Ca2+ in hearts of cardiomyopathic hamsters. Am J Physiol. 1993;264:H2188–97. 10.1152/ajpheart.1993.264.6.H2188 8322950

[61] Baines AD, Ross BD (1984). Gluconeogenesis and phosphate reabsorption in isolated lactate- or pyruvate-perfused rat kidneys. Miner Electrolyte Metab.

[62] Mather A, Pollock C (2011). Glucose handling by the kidney. Kidney Int.

[63] Garcia-Alvarez M, Marik P, Bellomo R (2014). Sepsis-associated hyperlactatemia. Crit Care.

